# *Placodus* (Placodontia, Sauropterygia) dentaries from Winterswijk, The Netherlands (middle Anisian) and Hünfeld, Hesse, Germany (late Anisian) with comments on ontogenetic changes

**DOI:** 10.1007/s12542-022-00614-w

**Published:** 2022-03-22

**Authors:** Nicole Klein, Tania Wintrich, Hans Hagdorn, Dave Spiller, Herman Winkelhorst, Gerard Goris, Torsten M. Scheyer

**Affiliations:** 1 Paleontological Institute and Museum, University of Zurich, Karl-Schmid-Strasse 4, 8006 Zurich, Switzerland; 2 Institute of Geosciences, Paleontology, University of Bonn, Nussallee 8, 53115 Bonn, Germany; 3 Muschelkalkmuseum Hagdorn, Schloßstraße 11, 74653 Ingelfingen, Germany; 4 Dave Spiller, Liebrechtstrasse 44a, 46240 Bottrop, Germany; 5 Herman Winkelhorst, Molenstraat 14, 7121 CS Aalten, The Netherlands; 6 Gerard Goris, Kolmschotlanden 43, 7542 Enschede, The Netherlands

**Keywords:** Muschelkalk, Dentaries, Morphology, µct data, Taxonomy, Ontogeny

## Abstract

Two recently found dentaries from the Lower Muschelkalk of Winterswijk (The Netherlands) and from the Upper Muschelkalk of an outcrop in the vicinity of Hünfeld (Hesse, Germany) are studied and compared to lower jaws of placodonts. As a result, the here described specimens can be assigned to *Placodus* cf. *gigas*. However, this assignment should be regarded as preliminary due to the isolated nature of the material. More diagnostic material is necessary to validate this affiliation. A certain morphological variability in *P. gigas* dentaries that had been pointed out before is also obvious among the new material. *Placodus gigas* has a wide paleogeography and stratigraphic range and a revision of the material assigned to *P. gigas* with new methods is overdue but beyond the scope of the current paper. The dentary from Hünfeld is with about 4 cm preserved length the smallest so far known dentary of a *Placodus*. It provides interesting insights in morphological changes during ontogeny and reveals differences in trajectories when compared to dentaries of different ontogenetic stages of *Cyamodus hildegardis.*

## Introduction

The Lower Muschelkalk locality of Winterswijk in the Netherlands is unique among classical Muschelkalk localities because it produces not only a large amount of isolated remains, but also articulated and associated skeletons of marine vertebrates (Oosterink et al. 2003, 2021; Heijne et al. 2019; Voeten et al. 2019a), as well as tracks and trackways of terrestrial vertebrates (Oosterink 2009; Oosterink et al. 2021). Sediments in the Winterswijk quarry are assigned to the Vossenveld Formation, which is correlated to the Wellenkalk facies and is early to middle Anisian in age (Hagdorn and Simon 2010; Hagdorn et al. 2020a, b). The sediments indicate a shallow marine and near coastal environment, the latter is documented by periodically desiccated surfaces, as indicated by large polygons separated by mudcracks (Dülfer and Klein 2006).

The most common marine reptiles in Winterswijk are Eosauropterygia (Heijne et al. 2019): the pachypleurosaur *Anarosaurus heterodontus* (Rieppel and Lin 1995; Klein 2009, 2012; Klein and Sander 2019) and *Nothosaurus* spp. (Albers 2011; Klein et al. 2015; Voeten et al. 2019b). *Lariosaurus* is documented by only two skulls of which one is associated with some postcranial material (Klein and Albers 2009; Klein et al. 2016). Skeletons and isolated elements but no skulls so far of an early pistosauroid can also be regularly found (Sander et al. 2014; Klein et al. 2015; Klein 2019). Further faunal elements are the tanystropheid *Tanystropheus antiquus* (Wild and Oosterink 1984; Spiekmann et al. 2019) and the enigmatic *Eusaurosphargis* aff. *dalsassoi* (Klein and Sichelschmidt 2014; Scheyer et al. 2019), as well as several vertebrate ichnotaxa (summarized in Oosterink 2009). A recent overview on the geology and fauna of Winterswijk is provided in Voeten et al. (2019a) and Oosterink et al. (2021). As in other classical Muschelkalk localities, placodonts are less abundant in Winterswijk relative to Nothosauroidea and Pachypleurosauria.

In spite of a low abundance of placodonts (Neenan et al. 2019), the locality of Winterswijk provides a certain endemic diversity of that group with the placodontiform *Palatodonta bleekeri* (Neenan et al. 2013) and the placodont *Pararcus diepenbroeki* (Klein and Scheyer 2014; During et al. 2017) being so far unique to Winterswijk. Albers (2005) described a “*Cyamodus*-like placodontoid” premaxillary/maxillary fragment with four teeth and one yet not functional tooth visible. One large placodont rib from Winterswijk was included in a microanatomical and histological study (Klein et al. 2019). Oosterink (1978) shortly described and figured a dentary from Winterswijk, which he assigned to *Placodus antiquior*. This specimen from Winterswijk was also mentioned and figured in Rieppel (1995:Fig. 34). Furthermore, several isolated teeth displaying the typical “crushing tooth morphology” indicating durophagous specialization of placodonts (Rieppel 2002), had been found over the years in Winterswijk. They are assigned to *Placodus gigas* as well (Oosterink 1978; Oosterink et al. 2003). Oosterink et al. (2003) also figured an isolated grasping tooth and assigned it to to cf. *Paraplacodus* sp., which would represent one of the few occasions of the genus outside the Alpine Triassic (e.g., Rieppel 2000a, b)*.*

*Placodus antiquior* was erected by Huene (1936) on the basis of a skull and a fragmentary dentary from the Lower Muschelkalk from the Schaumkalk of Freyburg/Unstrut. Subsequently over the years, other remains of *Placodus* from the Lower Muschelkalk have been assigned to *Placodus antiquior*, before the taxon was identified as a junior synonym of *P. gigas* (Rieppel 1995). According to Rieppel (1995), the morphological differences between *P. gigas* and *P. antiquior* are negligible and the erection of *P. antiquior* reflects “a general tendency of earlier authors to separate taxa from the Lower and Upper Muschelkalk for stratigraphic rather than morphological reasons” (Rieppel (1995:11).

In the Upper Muschelkalk of the central Germanic Basin, placodont remains are relatively rare, too. All remains had been assigned so far to *Placodus gigas* (summarized in Rieppel 1995). According to Rieppel and Hagdorn (1997), the range of *Placodus gigas* in the Upper Muschelkalk is restricted to its lower part up to the *evolutus* biozone, whereas *Cyamodus* reaches up to the Muschelkalk top. The locality Müllersrain near Hünfeld (Hesse, central Germany; Kramm et. al 2020) from where the here described small dentary originated, conforms to the upper part of the general range of *Placodus* within the Upper Muschelkalk. According to the finder (WG) and Manfred Schulz, Großenlüder (pers. comm. 2021 with HH), the quarry exposed a rather faulted section of a few meters thickness. The relatively lime-dominated facies with very rare ceratites in its upper part suggests a position in Folge m7b and is thus late Anisian in age (Hagdorn et al. 2020a, b). The depositional environment was shallow marine with bivalve-dominated mudgrounds. The lack of ceratites in the lower part of the small outcrop may be explained by adverse conditions for ceratite preservation in the lime-dominated facies.

The aim of this study is to describe two recently found dentaries of *Placodus* cf. *gigas* from the Lower Muschelkalk of Winterswijk, The Netherlands as well as a small (4.18 cm) *Placodus* cf. *gigas* dentary from the Upper Muschelkalk of Hünfeld (Hesse, Germany). We furthermore give a comprehensive description of the dentary from Winterswijk that was already mentioned before (Oosterink 1978; Rieppel 1995). The morphology is studied with the help of µct data. The material is compared to placodont dentaries with an emphasis on ontogenetic changes, morphological variability, and taxonomical affinities.

## Materials and methods

**Fig. 1:**
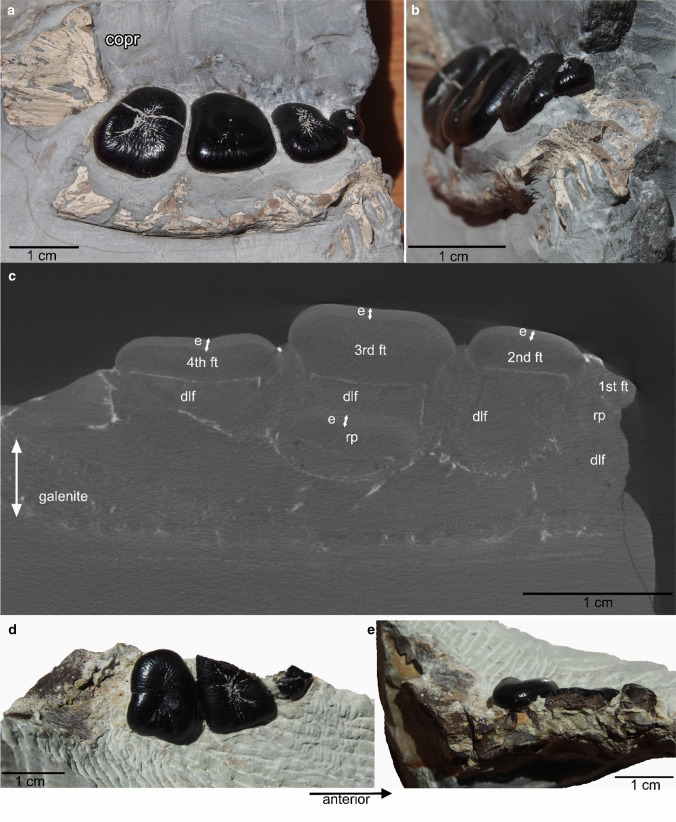
Left dentary MFW 20784 from the locality of Winterswijk (Lower Muschelkalk, middle early Anisian) depicting the complete marginal tooth row of four crushing teeth. **a** In medioocclusal view. Note the laterally shifted coronoid process. **b** In anterior view. Note the well-preserved mandibular suture and the dorsally exposed 2nd crushing tooth. **c** µct picture of MFW 20784 in dorsoventral view. Note the thick enamel of the functional teeth and the 3rd replacement tooth (white double arrows). The teeth are fused to a thin bony layer (septum), which separates the functional tooth from the dental lamina foramina. Note the large sediment filled cavities for the development of the replacement tooth below the 1st and 2nd tooth. Below the 3rd tooth an already well-developed replacement tooth is visible. The cavity below the 4th tooth is sediment filled and smaller. The “white lines” represent bone. The galenite layer on which the dentary is positioned is well visible. **d** Incomplete left dentary RGM.1333565 from the locality of Winterswijk (Lower Muschelkalk, middle early Anisian) in occlusal view depicting one complete and two incomplete teeth. **e** same specimen in dorsoventral view, exposing the thick enamel of the 1st and 2nd tooth, the conical shape of the 1st tooth, and the replacement tooth below the 1st tooth. Abbreviations: *copr* coronoid process; *dlf* dental lamina foramen (i.e., the cavity in which the replacement tooth develops, Rieppel 2001); *e* enamel; *ft* functional tooth; *rp* replacement tooth.

The left dentary MFW 20784 is stored in De Museum fabriek, Winterswijk. It was found in 1978 by Teun van Manen from Nieuwkoop, The Netherlands (Oosterink 1978). Unfortunately, there is no documentation of the find situation or from where exactly in the quarry it came. The division into sedimentological layers used still today (Oosterink 1986) was not established yet in 1978. The Vossenveld Formation (Hagdorn and Simon 2010; Hagdorn et al. 2020a, b), i.e., the exposed Muschelkalk profile at the locality of Winterswijk, is today divided into 63 layers of which layer 1–39 had been defined by Oosterink (1986). Layers –11 to –1, 0 and 40 to 51 had been subsequently added over time (Maxwell et al. 2016; actual research by GG). MFW 20784 was embedded directly on a distinct layer of galenite that is about 1 cm thick (Fig. 1c). On its presumed field bottom side, the slab contains a ripple mark layer. The galenite horizon is very distinct and was assigned to layer 35 by Oosterink (1986). Due to actual sedimentological work in the quarry, the galenite layer is now assigned to layer 36 (pers. obs. GG). An additional inspection in the quarry by two of us (GG, HW) in July 2021 resulted in the agreement that MFW 20784 must have been originated from layer 36. The specimen was assigned to *Placodus* (*antiquior*) *gigas* and figured in Oosterink (1978), Rieppel (1995), Oosterink et al. (2003), and Neenan et al. (2019) but never described in detail.

**Fig. 2:**
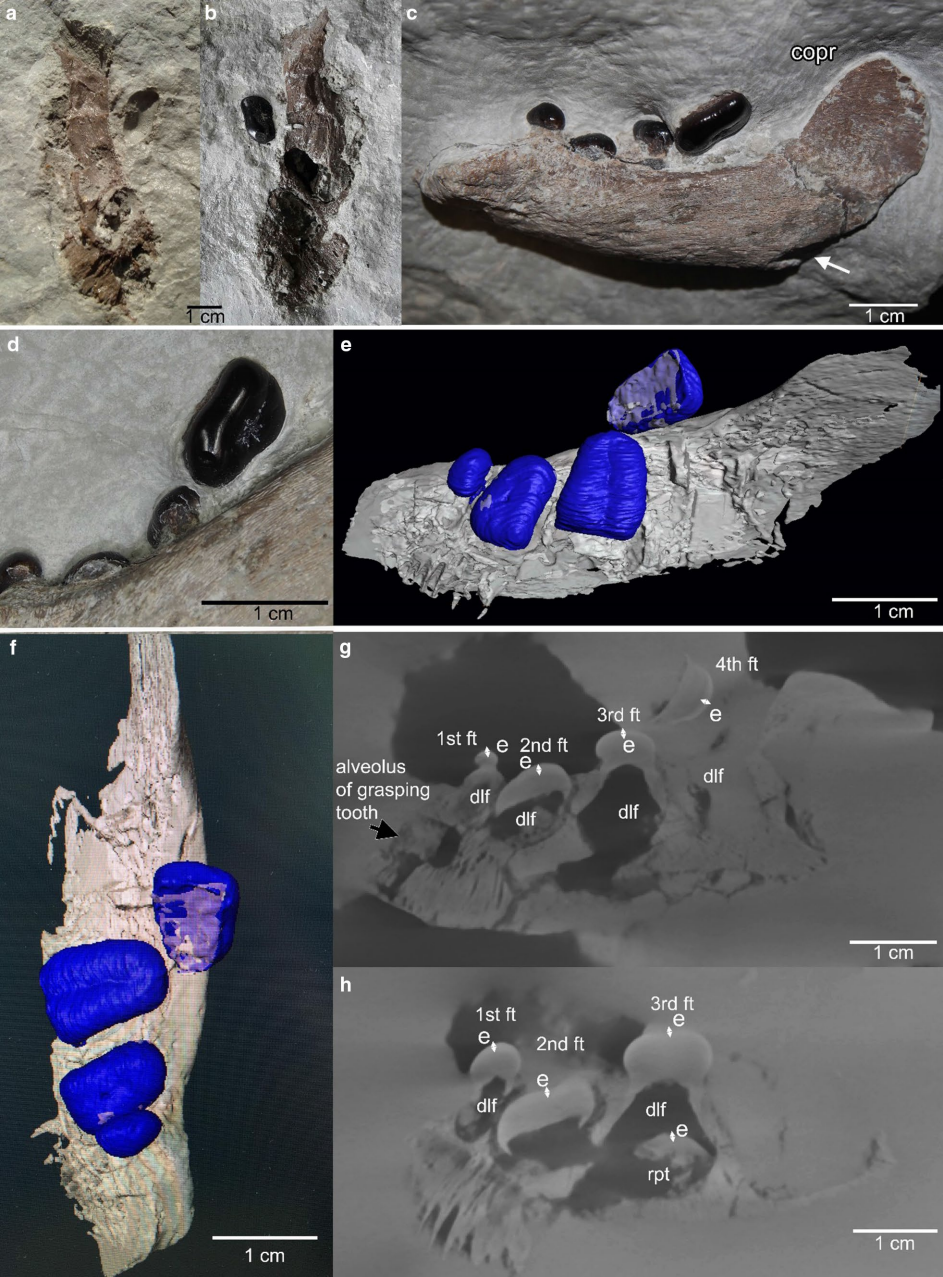
Left dentary DS.10.2015–921 from the locality of Winterswijk (Lower Muschelkalk, middle early Anisian) depicting the complete marginal tooth row of four crushing teeth. **a, b** Find situation. The specimen was split into a dorsal (**a**) and ventral (**b**) half that had been glued and mechanically prepared, resulting in the current state (**c**). **c** Left dentary DS.10.2015–921 in lateral view. Note the convex surface of the dentary and the rising coronoid process. Posteroventrally, the suture to the surangulare (arrow) is visible. The 1st tooth is smallest and conical, the following teeth are flat crushing teeth. The 2nd and 3rd tooth are in this view only partially exposed. The 3rd tooth has slightly disarticulated. The posterior-most, i.e., 4th, tooth in the row has disarticulated and flipped, pointing with its root now anterodorsally. **d** Detail of visible teeth. Note the typical worn pattern on the 4th tooth. **e**, **f** reconstruction of the dentary based on µct data in occlusal view (E) and lingual-occlusal view (F). The 2nd and 3rd crushing tooth are here well visible, exposing their rectangular shape. **g**, **h** µct picture of DS.10.2015–921 in labio-lingual view. Note the empty cavities (dlf) below the functional teeth. The last one is the only one filled by sediment. Only below the 3rd tooth is a replacement tooth visible. Abbreviations as in Fig. 1

Specimen DS.10.2015–921 was found in 2015 by one of us (DS), approximately 6–8 m above the main bone bearing horizon i.e., above layer 9 of Oosterink (1986). The exact layer could not be determined because the slab was found in the scree. The find horizon contained in addition to the dentary invertebrate burrows (*Rhizocorallium* sp.) and pyrite nodules. The presumed field bottom layer of the slab shows distinct ripple marks, indicative of belonging somewhere in the layers above 30 and below 40 (pers. obs. HW; GG), which are Bithynian in age (Herngreen et al. 2005). The slab containing the dentary was split into two halves, breaking the dentary roughly in the middle along its anteroposterior length, and resulting in a dorsal and a ventral half (Fig. 2a). The two parts were mechanically prepared by DS with a pneumatic engraving pen (HW-10) and surgeon tools. The dorsal part was freed from sediment and then glued on its ventral counterpart. Finally, mechanical preparation continued and uncovered the lateral and partially the occlusal side. The bone and the teeth were in a good state of preservation and show no damages or losses (Fig. 2). The original specimen DS.10.2015–921, is housed in the private collection of Dave Spiller, Bottrop, Germany. A cast and µct data are stored at the IGPB (IGPB R 627).

A very incomplete left dentary containing two incomplete teeth and one complete tooth in a row (RGM.1333565; Fig. 1d, e) was found in 2006 in layer 36. It was mechanically prepared by HW and donated to the collection of Naturalis in 2021.

MHI 2179 represents a very small right dentary from the Upper Muschelkalk of Hünfeld (Hesse, Germany) (late Anisian). It was found in the mid-1980s by Walter Koch, Großenlüder-Bimbach, in a small abandoned quarry at Müllersrain near Hünfeld (TK 25 5324, sheet Hünfeld; r. 35 52 62, h. 56 13 36) and donated to the Muschelkalkmuseum Ingelfingen in 2019. It is an important find because it represents the so far smallest dentary of a *Placodus* and documents changes in ontogeny when compared to larger specimens.

All specimens were measured with a caliper and photographed. µct scans of DS.10.2015–921, MFW 20784, and MHI 2179 were recorded with a v|tome|x s scanner manufactured by GE phoenix|X-ray (Wunstorf, Germany). The µct machine is operated by the Institute of Geosciences, Paleontology, at the University of Bonn (Bonn, Germany). The image stack of DS.10.2015–921, consisting of 418 images was imported into Aviso 8.0 and manually reconstructed by TW.

Lower jaws and/or dentaries assigned to *Placodus gigas* from the collections of the MFN, MHI, SMF, SMNS, and UMO were studied for comparison. Most of the material used for comparison was already listed in Rieppel (1995).

## Institutional abbreviations

**IGPB**, Institute of Geosciences, Paleontology, University of Bonn, Bonn, Germany; **MFW**, De Museumfabriek, Winterswijk, The Netherlands; **MFN**, Museum of Natural History, Berlin, Germany; **MHI**, Muschelkalkmuseum Hagdorn, Ingelfingen, Germany; **SMF**, Senckenberg Museum Frankfurt, Germany; **SMNS**, Staatliches Museum für Naturkunde Stuttgart, Germany; **TWE**, Museum TwentseWelle, now “De MuseumFabriek”, Enschede, The Netherlands; **UMO**, Urwelt Museum Oberbayern, Germany.

## Systematic palaeontology

Sauropterygia Owen, 1860

Placodontia Cope, 1871

***Placodus*** Agassiz, 1833

***Placodus gigas*** Agassiz, 1833

*Comment:* For dentaries of *Placodus,* the diagnosis of Rieppel (2000a, b:19) lists the following: […] dentary forming most of large coronoid process, lateral exposure of coronoid bone restricted; mandibular symphysis elongate, formed by dentaries and splenials; […]“.

*Placodus* cf. *gigas*

Figures 1–3

*Referred material.* Dentaries DS.10.2015–921, MFW 20784, and RGM.1333565 from the Lower Muschelkalk, early middle Anisian of the locality of Winterswijk (The Netherlands).

Figures 1–2

Dentary MHI 2179 from the Upper Muschelkalk, late Anisian of Hünfeld (outcrop Müllersrain, TK 25 sheet 5324 Hünfeld, Hesse, Germany).

Figure 3

### Description of Winterswijk material

MFW 20784 is a left dentary that measures 5.63 cm in preserved length and is preserved in lingual-occlusal view (Fig. 1a–c). The coronoid process is dorsally incomplete. Its preserved part has shifted lateroventrally in relation to the tooth row (Fig. 1a). What is visible from the bone appears rather thin. The dentary contains one very small and three large crushing teeth. The former is located anterior most in the marginal row (Fig. 1a–c). The dentary is anteriorly incomplete, the part in front of the 1st visible marginal tooth, i.e., the area of the diastema, has broken off, thus lacking the anterior part of the dentary with the frontal teeth. The already broad dentary is posteriorly expanding, which is at the height of the 3rd and 4th tooth. The distinct medial symphysis is anteriorly incomplete. It starts at the posterior end of the 2nd tooth. All preserved teeth are typical rectangular to trapezoidal (meaning the lingual side is longer than the labial one), flat, low-crowned crushing teeth. All teeth are wider (labiolingually) than long (mesiodistally) (Fig. 1a). The 1st tooth is the smallest. It is nearly reduced when compared to the size of the following teeth. The following teeth increase in size with the 3rd being the largest in the row and the 4th being somewhat smaller again (Fig. 1a). It is likely that the 1st tooth once was more conical, and is just worn down to its now flat condition. However, it not as worn down as in other specimens where the brown dentine is then visible (pers. obs. HH). It is positioned anterior and labiolingually central to the 2nd tooth. In all teeth, the enamel is thick, black and shiny (Fig. 1a–c). Except for the 3rd tooth, all teeth show a central, elongated labiolingually running depression and distinct rugosities on their occlusal surfaces (Fig. 1a, b), indicating that they had been in use for a while. The 3rd tooth is smooth and slightly convex maybe just replaced to a functional tooth and not long in usage at time of death.

The µct data provide insights into the inner structure of MFW 20784 (Fig. 1c). The teeth can be separated into a crown and root region and the enamel is well to distinguish from the dentine (Fig. 1c). The functional teeth are sitting in alveoli with the root fused to the bone. Thus, the anchorage of the teeth is ankylosed thecodont (Rieppel 2001). Below these alveoli of the functional teeth are cavities, i.e., the dental lamina foramina (Rieppel 2001:Fig. 1). Those cavities that do not contain a replacement tooth are hollow and are in MFW 20784 filled with sediment. Below the 1st and 3rd tooth is a replacement tooth developed. The replacement tooth below the 3rd tooth is far developed, already moving dorsally, being close to press out and replace the functional tooth (Fig. 1c; tooth replacement stage 3 of Neenan et al. 2014). Below the other functional teeth, the cavities are empty, now sediment filled, respectively. Thus, tooth replacement pattern is vertical (Rieppel 2001). Only one generation of replacement teeth is visible but not below every functional tooth.

DS.10.2015–921 is a left, anteriorly incomplete dentary that is prepared in lateral view. It contains four teeth and measures 7.5 cm in length (Fig. 2). The posterior part of the 3rd tooth is slightly dislocated in dorsal direction. The 4th tooth in the row is completely disarticulated and flipped about ~ 110°, exposing its root area (Fig. 2). The suture between the dentary and angular is posteroventrally visible but the angular is lost. It is deeply oval and encompassed ventrally and laterally by the dentary (Fig. 2c, arrow). Behind the last tooth the dentary ascends in a slight angle (~ 45°) to the coronoid process. It cannot be clarified if the posterodorsal process of the dentary covers most of the lateral surface of the coronoid process as is the condition in *Placodus gigas* (Rieppel 1995). In DS.10.2015–921 no distinction between coronoid and posterodorsal process of the dentary can be made. Although incomplete, the coronoid process seems to be neither very steep nor very high when compared to other *Placodus gigas* dentaries. The lateral surface of the dentary is slightly convex, depicting several large and small foramina that are in anteroposterior direction elongated and situated below the 2nd and 3rd tooth (Fig. 2c). The bone surface is rugose (Fig. 2c). The dentary is broadest in dorsal view at the height of the 3rd tooth. The anterior part of the dentary is at the height of the boundary of the 1st and 2nd tooth constricted. The posterior end of the medial symphysis starts at the middle of the 2nd tooth (Fig. 2e). The anterior part of the dentary is incomplete, missing the anterior most part and the anterior chisel-shaped dentary teeth. The tooth row of the crushing teeth is lingually oriented, slightly dipping with the occlusal surface in this direction. In occlusal view, the 2nd to 4th teeth are typical rectangular to trapezoidal (meaning the lingual side is longer than the labial one), flat, low-crowned crushing teeth; the 1st one is round and conical with a somewhat higher crown (Fig. 2c, d). It is positioned anterior and labiolingually central to the 2nd tooth. The 2nd and 3rd crushing tooth are wider in labiolingual direction than long in anteroposterior direction. The 4th crushing tooth is labiolingually wider than than mesiodistally long (Fig. 2d). The 3rd tooth is the largest in the row, the 4th is second largest, the 2nd is the smallest of the crushing teeth and the 1st one is smallest in the entire row (Fig. 2) but it is not as reduced as in other specimens. The enamel is thick, black and shiny (Fig. 2). The three posterior crushing teeth (2nd to 4th) have a central, elongated labiolingually extending depression on their occlusal surfaces (Fig. 2d–f), indicating that they had been in use for a while. Based on the µct pictures the teeth can be separated into a crown and root region and the enamel is well to distinguish from the dentine (Fig. 2g, h). The functional teeth are sitting in alveoli with the root fused to the bone. Thus, the anchorage of the teeth is ankylosed thecodont (Rieppel 2001). Below the alveoli of the functional teeth, cavities are visible, i.e., the dental lamina foramina (Rieppel 2001). Those cavities that do not contain a replacement tooth are hollow (1st, 2nd) or filled with sediment (4th) (Fig. 2g, h). Below the 3rd tooth a replacement tooth can be identified (tooth replacement stage 2 to 3 of Neenan et al. 2014) and tooth replacement is identified as being vertical (Rieppel 2001) (Fig. 2g, h). Ventro-anteriorly, straight rectangular alveoli of the two front teeth are visible. They dip below the alveolus of the 1st crushing tooth (Fig. 2g).

RGM.1333565 consists of two incomplete and one complete tooth, aligned in a row belonging to a left dentary (Fig. 1d, e). The specimen measures as preserved 2.7 cm. Bone is only as “impression” visible. Only the lingual half of the 1st tooth is preserved, exposing the thickness of the enamel and a fragmentary replacement tooth below (Fig. 1d, e). The 1st tooth is the smallest tooth and was likely conical (Fig. 1e). The anterolabial corner of the 2nd tooth has broken off, exposing here the thick enamel, too. The 2nd tooth appears to have been rectangular/square-shaped and the 3rd tooth is trapezoidal (meaning the lingual side is longer than the labial one). Both are of a similar size and show thick, black and shiny enamel (Fig. 1e). Each of the two crushing teeth displays a central, elongated labiolingually extending depression and distinct rugosities on their occlusal surfaces (Fig. 1d, e), indicating some usage. Contrary to MFW 20784 and DS.10.2015–921, RGM.1333565 had only three teeth (Fig. 1e). It is not clear if a 4th posterior-most tooth had been there and was lost during preservation.

### Description of MHI 2179

**Fig. 3:**
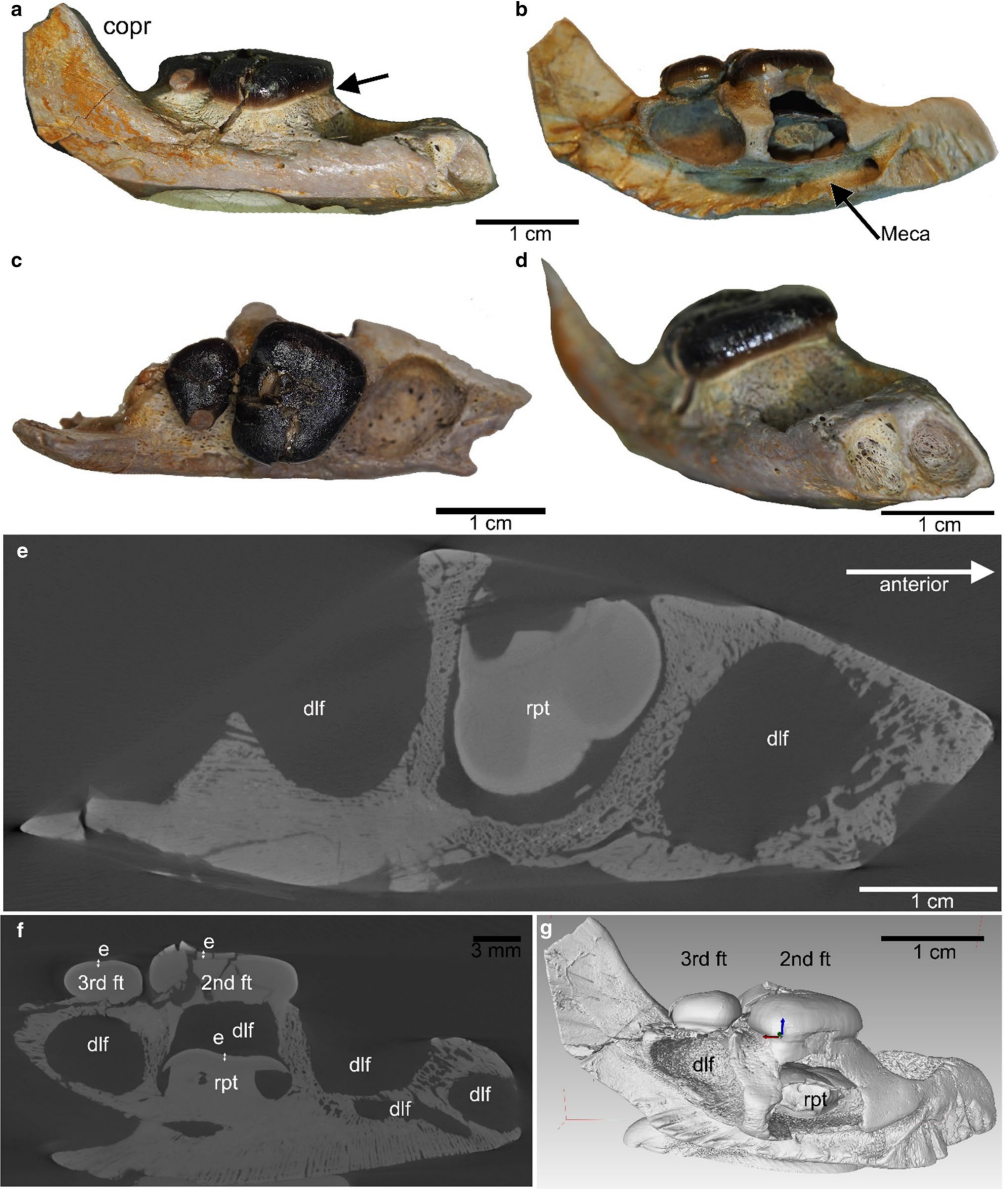
Right dentary MHI 2179 from Hünfeld (Upper Muschelkalk, late Anisian). **a** In lateral view, depicting a narrow, angled lateral side, the slightly rising coronoid process and the two preserved functional teeth and the empty alveoli of the 1st crushing tooth and the two lost grasping teeth. The suture with which the functional tooth is fused to the bone (septum) is well visible (arrow) as is the enamel dentine boundary and the constriction separating the crown from the low root (arrow). **b** In medial view, showing from dorsally to ventrally: the two preserved teeth, their root region, the cavity below the functional tooth for the development of the replacement tooth (dlf), the replacement tooth below the 2nd functional tooth, and the Meckelian canal with two large foramina. **c** In occlusal view. Note the numerous foramina at the bottom of the alveolus of the 1st-lost-crushing tooth. **d** In anterior view, displacing the two alveoli of the lost frontal grasping teeth. **e** µct picture of the occlusal view, depicting the empty cavities (dlf) of the 1st and 3rd crushing tooth and the replacement tooth below the 2nd crushing tooth. Note the high porosity of the bone in all pictures. **f** µct picture in lateral view. **g** VGL Studio Max 3D picture in lateral view. Abbreviations as in Fig. 1, Meca, Meckelian canal.

MHI 2179 is a right, anteriorly incomplete dentary that displays one empty alveolus, two functional teeth, and one replacement tooth (Fig. 3). It is very small, measuring 4.16 cm in length. In lateral view, the posterior part of the dentary rises smoothly in a rough 45° angle, forming the relatively high but posteriorly incompletely coronoid process. A coronoid cannot be identified and is likely broken off. The lateral side is not convex but descends from the occlusal surface down medioventrally (Fig. 3a). Except for the foramina the bone surface is smooth. µct data of MHI 2179 reveal rather spongious bone (Fig. 3e, f). The lingual side of the dentary exposes the part that carries the functional teeth and below these, the cavities or caverns for the replacement teeth, i.e., the dental lamina with its foramina are visible (Rieppel 2001). These cavities are in MHI 2179 additionally well exposed due to the loss of the splenial (Fig. 3b). Anteriorly, two alveoli are visible, of which the lingual one is the deeper and larger one. These had carried the now lost frontal teeth. Directly along the lateral margin of these alveoli are three large foramina located and below dozens of smaller scattered foramina are visible. MHI 2179 contains three crushing teeth. The anterior crushing tooth is lost, now showing its empty alveolus. The 2nd crushing tooth is the largest. The 3rd tooth is very small (Fig. 3). Both preserved teeth are trapezoidal in dorsal view with being lingually broader than labially. The form of the empty alveolus suggests a similar shape for the lost 1st tooth. The crushing teeth are flat and low-crowned with thick, black and shiny enamel (Fig. 3a–d). A grinding pattern is not observed on the surface of the two preserved functional teeth (Fig. 3c). In MHI 2179 the 1st preserved tooth was a large crushing tooth. It is unlikely that another small tooth —as in DS.10.2015–921 and MFW 20784— was present anterior, because of space, i.e., the alveoli of the frontal grasping teeth start already in this area (Fig. 3c, d). The dentary is broadest in dorsal view at the height of the 2nd tooth. In front of the anterior most crushing tooth or its alveolus, respectively, the lateral side of the dentary is weakly constricted. The diastema between the marginal crushing teeth and the anterior grasping teeth is very narrow. The posterior end of the medial symphysis starts at the posterior end of the 1st tooth (Fig. 3b). The tooth row of the crushing teeth is lingually oriented, slightly dipping with the occlusal surface in this direction as well. Below the 2nd tooth is a well-developed replacement tooth visible, proofing a vertical replacement pattern (Fig. 3b). The replacement tooth represents tooth replacement stage 3 of Neenan et al. (2014). It is larger when compared to its preceding functional tooth (2nd tooth in the row; Fig. 3b), which is according to Rieppel (2001) normal in postembryonic growth. The cavity below the 3rd tooth is empty. If it is empty because the replacement tooth has fallen off or because a replacement tooth has not yet developed is unknown. The replacement tooth cavity below the 1st tooth is closed by the bony wall forming the symphyseal suture (Fig. 3b). The canal that had held the Meckel’s cartilage is well exposed, which is again due to the loss of the splenial. It extends along the entire tooth bearing part of the lingual side of the dentary and ends anteriorly in a large foramen below the cavity of the 1st replacement tooth. Below the bony wall that is between the 2nd and 3rd alveolus is another large foramen. Due to the lack of the splenial, the anchorage of the 2nd functional crushing tooth (i.e., the 1st preserved tooth) is well exposed and clearly shows that the root is fused to the bone (Fig. 3a). Thus, tooth anchorage is ankylosed thecodont. This specimen also nicely shows the low root that is separated from the crown by a distinct constriction. The root is devoid of enamel (Fig. 3a). One generation of replacement teeth is visible. The µct data of MHI 2179 support the observations made by classical morphological study and additionally reveal a very porous bony structure (Fig. 3e).

## Comparison

### Winterswijk dentaries

The three described dentaries from Winterswijk share the flat and rectangular teeth typical for *Placodus gigas* (Rieppel 1995, 2000a, b, 2002). MFW 20784 and DS.10.2015–921 further have the steep and broad coronoid process preserved, also indicative for *Placodus gigas* (Rieppel 1995, 2000a, b). Beyond these similarities the dentaries from Winterswijk show some morphological variation. DS.10.2015–921 is the largest representative of the here described specimens but its teeth are relatively smaller. The occlusal width of the dentary is in relation to the length of the dentary not as broad as in MFW 20784 and RGM.1333565. The 1st tooth of DS.10.2015–921 is relatively larger when compared to the reduced 1st tooth of MFW 20784 and RGM.1333565. Its conical shape compared to the flat condition in the latter two, however, can be the result of being not worn down yet. The teeth of DS.10.2015–921 are rectangular, i.e., they are labiolingually wider and not as long (in anteroposterior direction) when compared to the more squared-shaped and trapezoidal teeth of MFW 20784 and RGM.1333565. The dentaries themselves cannot be compared because the bone is only well preserved in DS.10.2015–921. The more massive appearance of dentary DS.10.2015–921 when compared to the other two remains from Winterswijk is likely the result of preservation. The symphyseal suture ends in DS.10.2015–921 at the height of the middle of the 2nd tooth but in MFW 20784 at the posterior end of the 2nd tooth. In mesial direction, this suture reaches in DS.10.2015–921 well beyond the 1st tooth. Due to incompleteness, the extension of this suture is not visible in MFW 20784; it is traceable as far as to the 1st tooth but then the dentary ends in a breakage.

### Winterswijk dentaries compared to MHI 2179

Despite the smaller size of MHI 2179, the overall morphology concurs in the dentaries from Winterswijk and MHI 2179. The main differences relate to the lower number of crushing teeth (three vs. four) and the less high and not convex lateral side of the dentary in MHI 2179 when compared to the dentaries from Winterswijk. The bone is spongious in MHI 2179 when compared to that of DS.10.2015–921. The lateral side of the dentary MHI 2179 is narrow and straight to angled (dipping in medioventral direction). In MHI 2179, the 2nd, i.e., the middle crushing tooth is the largest tooth whereas in the Winterswijk dentaries this is the 3rd tooth. The last crushing tooth is the smallest in MHI 2179 but in the Winterswijk specimens the 2nd tooth in the row is the smallest of the large crushing teeth, not considering the reduced 1st tooth in the marginal row.

### Comparison of here described dentaries to those of other placodonts

For paleogeographic and stratigraphical reasons, the comparison is restricted to *Paraplacodus broilii*, *Placodus gigas*, and *Cyamodus* spp. (see Rieppel 2000a, b). *Paraplacodus broilii* differs in tooth morphology from the material from Winterswijk and Hünfeld by having much smaller round and conical/hemispherical high-crowned teeth and a much lower coronoid process (Rieppel 2000b). Gere et al. (2020) pointed out a shoulder-like depression on the occlusal surface of the crushing teeth of *P. broilii*, which differs from the occlusal pattern in the Winterswijk teeth showing central, elongated labiolingually extending depressions and rugosities that split to both directions. Specimen MHI 2179 does not show any grinding pattern. The single frontal tooth assigned to cf. *Paraplacodus* sp. by Oosterink et al. (2003) is undiagnostic and its assignment is regarded doubtful herein.

Affinities of the placodont dentaries from Winterswijk and that of Hünfeld to teeth of *Cyamodus* spp. can be excluded as well. Crushing teeth of *Cyamodus* spp. are round-oval, higher crowned, and have a large central radially striated occlusal depression (Kuhn-Schnyder 1959; Gere et al. 2020; Brignon 2021). Further, the morphology of the dentary differs (see Rieppel 1995). The premaxillary/maxillary fragment with four teeth and one yet not functional tooth described by Albers (2005) as “*Cyamodus*-like placodontoid” from Winterswijk likely belongs to the ichthyopterygian *Tholodus schmidi* and not to a placodont. Arguments supporting this are the irregularly spaced bulbous teeth capped by a dark brown crown (not black and shiny!) displaying longitudinal wrinkled enamel (see Albers 2005:Fig. 1 vs. description in Sander and Mazin 1993). However, the root area is not exposed on the photo and it cannot be clarified if the root is high and if the dentine is infolded in the specimen (Albers 2005). Unfortunately, the original specimen currently cannot be located (pers. com. Ch. de Jong, TWE) and thus is not available for detailed re-examination. The specimen is in any case, for the above-mentioned differences in tooth morphology, very different from the here described dentaries from Winterswijk and Hünfeld.

The crushing teeth of *Placodus inexpectatus* from the Anisian of China are not well exposed (Jiang et al. 2008) and thus difficult to compare. The slender dentary of *P. inexpectatus* might be the result of preservation/preparation. The preserved parts of the dentaries from Winterswijk and the stratigraphically younger dentary MHI 2179 fit in general well to the overall description of the lower jaw, especially that of the dentary, of *Placodus gigas* (Drevermann 1933; Rieppel 1995). A certain variability of morphological features is typical for *P. gigas* as was already pointed out by Rieppel (1995). For example, Rieppel (1995:23) mentioned the “variation in tooth counts seen in the posterior dentition of the lower jaw, with three large tooth plates being the norm”. However, “in a number of individuals, a distinctly smaller and rounded” (4th; i.e., here the 1st) crushing tooth “is positioned immediately in front of the [three] large dentary crushing teeth”. The three dentaries from Winterswijk show such a small rounded tooth as described above, whereas MHI 2179 does not. Rieppel (1995) discussed a possible late ontogenetic addition of this small crushing tooth but pointed out that out of 24 dentaries seven, which span however, the entire size range, show four crushing teeth. Thus, four dentary crushing teeth can occur in small individuals as well as in larger individuals and ontogeny alone might not be the reason for the varying number of teeth (Rieppel 1995). In the study of Rieppel (1995), MFW 20784 (which has four crushing teeth) represented the smallest available specimen. Some additional observations made here refer to the size and position of this 1st tooth. If present, this 1st tooth is conical (vs. the flat crushing teeth) and usually very small i.e., reduced (~ ¼ of the size of the following crushing tooth (see e.g., MHI 986, MHI 1055, SMNS 58,021, Drevermann 1933: plate 2, Fig. 2a). However, in DS.10.2015–921 and MFN 85 this 1st tooth is relatively large (half the size of the following tooth). The position of the 1st tooth can vary as well. It is located in the middle of the succeeding tooth (e.g., MHI 1055; DS.10.2015–921, MFW 20784) or labial to it (e.g., SMNS 58021, MHI 986, Drevermann 1933: Tafel 2 Fig. 2a).

A depression at the anteroventral margin of the dentary, approximately below the first large crushing tooth is present in some specimens. According to Rieppel (1995:23), the splenials meet here in an interdigitating medioventral suture behind the mandibular symphysis, thus contributing to the anteroposterior elongation of this symphysis. This depression however, is not identified in MHI 2179, DS.10.2015–921, and SMNS 58021, which are all less than < 8 cm in length (the area is not preserved in MFW 20784 and RGM.1333565) but occurs in most of the large specimens (> 10 cm in length) where this area is preserved (e.g., Drevermann 1933: Tafel 2 Fig. 2a; SMF R 4112; MHI 1055). It is less pronounced in MHI 986 and an unnumbered specimen from a locality in the vicinity of Bayreuth stored in the MFN collection. Both the latter specimens are of a similar large size as SMF R 4112 and MHI 1055. The coronoid process appears in some specimen’s steeper (ascending angle of about 75°) than in others (ascending angle of about 45°), which can be related to preservation and orientation of the isolated dentaries. However, when comparing SMNS 54436 with the holotype specimen described by Drevermann (1933: plate 2, Fig. 2a), it is clear that this is not solely related to orientation because both are more complete in their posterior region and orientation of the dentary is set in the sediment. Ontogeny can be excluded as well because both are of a similar size.

The extension of the mandibular symphysis is variable, too. In some specimens it extends from the anterior most crushing tooth or from the area of the diastema back to the posterior crushing tooth whereas it ends anteriorly in others. The same applies to the length of the diastema between the marginal crushing and the anterior grasping teeth: some have a short, others a longer diastema. However, due to the incompleteness of the anterior dentaries both is difficult to measure in most of the specimens.

The shape of the crushing teeth, also in general awkward, is variable, too. They can be square-shaped (e.g., SMF R 4112, Drevermann 1933: plate 2, Fig. 2a; MFW 20784, RGM.1333565, and MHI 2179) or rectangular to trapezoidal (SMNS 58021, MHI 1055, MFN Freyburg Nr 10/1913, Drevermann 1933: plate 2, Fig. 2a). The teeth of DS.10.2015–921 are more rectangular instead of square-shaped, being distinctly wider than long.

In all large *P. gigas* dentaries (> 10 cm), the posterior-most crushing tooth is the largest (Rieppel 1995). In the smaller Winterswijk dentaries (< 8 cm), the 3rd crushing tooth i.e., the second last is the largest crushing tooth. This is most distinct in DS.10.2015–921 but less clear in MFW 20784 and RGM.1333565 where the last and second last tooth are of a nearly similar size. In MHI 2179 the second last crushing tooth is the largest in the row but here the last one is distinctly smaller, which is likely due to ontogeny.

## Discussion

### Taxonomical assignment of the here described dentaries

Following the description of dentaries by Drevermann (1933) and Rieppel (1995, 2000a, b) for *Placodus gigas*, as well as when comparing dentaries assigned to *Placodus gigas* in the above-mentioned collections, the Winterswijk dentaries and the dentary from Hünfeld can be assigned to the genus *Placodus* based on the shape and size of their crushing teeth and the steep and broad coronoid process (where preserved). Because the genus so far contains only *P. gigas* we assign the here described material preliminary to *Placodus* cf. *gigas* until more complete and diagnostic material will be found. This is because the material from Winterswijk and the isolated dentary from Hünfeld are fragmentary and originate from different stratigraphical levels. More complete and diagnostic finds are necessary to fulfill the criteria from the diagnosis of *Placodus gigas* (Rieppel 1995, 2000a, b). Further, a revision of all the Muschelkalk material assigned to *Placodus gigas* with, for example, the help of µct data is advisable for future research. It must be clarified if the observed morphological differences between the dentaries described above are taxonomically relevant or reflect intraspecific variability. Muschelkalk material assigned to *P. gigas* is spanning a wide stratigraphical and geographical range (e.g., summarized in Rieppel 1995, 2000a, b, 2002) and it is conceivable that more than one taxon existed. However, such an analysis is beyond the scope of this paper. Our results confirm the taxonomical assignment for placodont material from Winterswijk of earlier studies (Oosterink 1978; Rieppel 1995).

### Comments on ontogenetic changes in dentition

MHI 2179 represents so far the smallest dentary of a *Placodus* described. The three dentaries from Winterswijk are also small (< 8 cm) when compared to other Muschelkalk dentaries (> 10 cm; Rieppel 1995). Besides its small size, several features point to an early ontogenetic stage of MHI 2179 when compared to the Winterswijk dentaries and other specimens of *P. gigas*. The bone tissue in MHI 2179 is rather spongious when compared to that of DS.10.2015–921, maybe also indicating an earlier ontogenetic stage by a lesser degree of ossification. Differences in the overall morphology of MHI 2179 and other, larger specimens, suggest a change in morphology of the dentary during ontogeny. In lateral view, MHI 2179 is not very high and its lateral surface is angled and dips in lingual-ventral direction instead of being broad and convex as in larger specimens. The ventral depression where the splenials meet is not visible in MHI 2179 but in larger specimens. The length of the medial symphysis and the length of the diastema is in MHI 2179 short when compared to their extension in larger specimens. As Rieppel (1995) already stated, the presence of a 4th (reduced) anterior most tooth in the row of marginal crushing teeth is not a good indicator for ontogeny. This is also reflected in the here studied material: MHI 2179 has three marginal/crushing teeth whereas MFW 20784 and DS.10.2015–921 have four. It cannot be clarified if RGM.1333565 has had four or three marginal teeth. In large specimens (> 10 cm) of *P. gigas*, the largest crushing tooth is always the last one in the row (Drevermann 1933; Rieppel 1995). In this position, directly anterior and medial to the coronoid process, it provides optimal crushing forces together with the dentition from the upper jaw (Rieppel 2002). In MHI 2179, the second last is the largest and the last one is the smallest tooth. In DS.10.2015–921 and MFW 20784, also the second last tooth is the largest one. In both specimens, however, the last crushing tooth in the row is larger as in MHI 2179 but still not as large as or larger than the second last. DS.10.2015–921 and MFW 20784 are roughly double in length as MHI 2179 but they are still smaller when compared to other dentaries of *P. gigas* (Drevermann 1933; Rieppel 1995). Thus, it is conceivable that the position of the largest tooth changes during ontogeny from a more anterior position (second last tooth) to the posterior-most position (last tooth). This might be related to limited space in the posterior dentary in ontogenetic younger individuals and/or to different crushing forces/biomechanics during ontogeny. However, due to regular tooth replacement, the last tooth of earlier ontogenetic stages could easily be enlarged during ontogenetic growth simultaneously with the growing dentary until the condition (i.e., last tooth becomes largest). This is visible in DS.10.2015–921 and MFW 20784, as well as in large *P. gigas* specimens. This process is also already visible in MHI 2179 where the replacement tooth below the 2nd functional tooth is larger than its precursor. Likely, the angle of the coronoid processes also becomes steeper during ontogeny.

Comparing our observation on ontogenetic changes in the dentition of MHI 2179 to those of *Cyamodus hildegardis* made by Kuhn-Schnyder (1959), some similarities but also differences are obvious. According to Kuhn-Schnyder (1959), the teeth in earlier ontogenetic stages in *Cyamodus hildegardis* are more pointed when compared to older ontogenetic stages, which might be related to the fact that the teeth of this individual simply had not been long in use, i.e., worn down before it died. In any case, the teeth of MHI 2179 are not more pointed when compared to those of larger specimens. The mandibular symphysis is getting longer during ontogeny in *Cyamodus hildegardis* and *P. gigas,* whereas the diastema is getting shorter in *Cyamodus hildegardis* but longer in *P. gigas*.

In *Cyamodus hildegardis* the number, spacing, position, and size of the teeth on the dentary changes during ontogeny, whereas shape stays the same (Kuhn-Schnyder 1959; Neenan et al. 2014; Wang et al. 2019a). *Cyamodus hildegardis* shows a clear reduction in the number of mandibular teeth, which is accompanied by a simultaneous increase in the size of the remaining teeth from juvenile to adult individuals. The number of mandibular teeth does not change in *P. gigas* but the size relation of crushing teeth changes from the second last tooth being the largest to the last tooth in row becoming largest. With every replacement, a reinforcement of the posterior functional teeth is hypothesized, since they are closest to the center of rotation i.e., the jaw joint. Our observations thus yielded different ontogenetic trajectories in dentition when comparing *Cyamodus hildegardis* and *P. gigas*. However, one has to consider that the smallest dentary of *C. hildegardis* is distinctly smaller (~ 1 cm) when compared to the small dentary assigned to *Placodus* cf. *gigas* (MHI 2179; 4.16 cm). The *Cyamodus* material described by Kuhn-Schnyder (1959), thus likely represents even an earlier ontogenetic stage than the latter. MHI 2179 is in an ontogenetic (i.e., when considering size) and morphological perspective closer to the adult condition and ontogenetically influenced changes are less pronounced when compared to *Cyamodus hildegardis*. Wang et al. (2019b) described a positive allometric growth of the posterior portion of the skull for the Chinese *Sinocyamodus xinpuensis* when comparing an adult and a subadult individual. This observation of Wang et al. (2019b) for *S. xinpuensis* fits to our interpretation of a reinforcement of the posterior part of the dentary and the posterior functional teeth.
